# Fabrication of on-chip probes for double-tip scanning tunneling microscopy

**DOI:** 10.1038/s41378-020-00209-y

**Published:** 2020-11-02

**Authors:** Maarten Leeuwenhoek, Freek Groenewoud, Kees van Oosten, Tjerk Benschop, Milan P. Allan, Simon Gröblacher

**Affiliations:** 1 Kavli Institute of Nanoscience, Department of Quantum Nanoscience, Delft University of Technology, Lorentzweg 1, 2628CJ Delft, The Netherlands; 2 Leiden Institute of Physics, Leiden University, Niels Bohrweg 2, 2333CA Leiden, The Netherlands

**Keywords:** Nanoscale devices, Electronic properties and materials

## Abstract

A reduction of the interprobe distance in multiprobe and double-tip scanning tunneling microscopy to the nanometer scale has been a longstanding and technically difficult challenge. Recent multiprobe systems have allowed for significant progress by achieving distances of ~30 nm using two individually driven, traditional metal wire tips. For situations where simple alignment and fixed separation can be advantageous, we present the fabrication of on-chip double-tip devices that incorporate two mechanically fixed gold tips with a tip separation of only 35 nm. We utilize the excellent mechanical, insulating and dielectric properties of high-quality SiN as a base material to realize easy-to-implement, lithographically defined and mechanically stable tips. With their large contact pads and adjustable footprint, these novel tips can be easily integrated with most existing commercial combined STM/AFM systems.

## Microscopy: On-chip probes for double-tip scanning tunneling microscopy

A procedure has been developed for fabricating on-chip double-tip devices using two mechanically fixed gold tips with nanometer-scale separation for application in scanning tunneling microscopy (STM) systems. Reducing the inter-probe distance in multi-probe and double-tip STM (using two tips simultaneously in tunneling) to the nanometer scale has long been a technically difficult challenge. But a team headed by Milan Allan at Leiden University and Simon Gröblacher at Delft University of Technology, The Netherlands has succeeded in devising a fabrication procedure for integrated double-tip devices with a tip separation of around 35 nanometers. The team used the excellent mechanical, insulating, and dielectric properties of high-quality silicon nitride as a base material to create simply implemented, mechanically stable tips. The authors believe that the large contact pads and adjustable footprint of the novel tips allow easy integration with most existing STM systems.

## Introduction

Scanning tunneling microscopy (STM) that uses two tips simultaneously, also called double-tip STM, relies on two individually driven metal wire probes brought into close proximity to locally probe the resistivity^1–3^ or access proposed electron correlations at the nanoscale^4–11^. Achieving tip separation down to the nanometer scale, a long-standing goal in multiprobe STM, has proven challenging and is limited by the radius of curvature of the two tips^12^ and requires sophisticated navigation routines^13,14^. Recently, multiprobe systems able to achieve tip separation down to 30 nm have emerged^1,13^ and have resulted in the first double-tip correlation measurements to date^15^.

These experiments, however, must undergo complicated alignment procedures and are limited to specialized STM setups. Here, we continue to build on earlier work^16^ to create a robust and easy-to-implement on-chip solution where both tips are integrated on a silicon chip. With our approach, given the joined nature of the tips, we can eliminate the need for an additional scanning electron microscope column^13,17–21^ for navigation and make this approach compatible with ultrastable compact Pan-type STM heads widely used for single-tip experiments^22^. Moreover, the millimeter-scale contact pads and adjustable footprint allow for easy integration in existing and commercially available STM systems. Existing on-chip scanning probes have already contributed to several techniques, such as parallel AFM^23^, scanning near field microscopy^24^, scanning Hall probes^25,26^, and scanning SQUID probes^27^. However, the development and use of integrated STM tips has been limited^28–31^. Recent proof-of-principle experiments have demonstrated that such *single-tip* probes can meet the stringent criteria for STM^29^ even under ultrahigh vacuum and low-temperature conditions^16^.

The main challenge in realizing multitip STMs is to minimize the tip-to-tip distance while maintaining the excellent stability required for prolonged in- and out-of-feedback measurements to obtain high-quality topographic and spectroscopic data. The double-tip devices presented in this letter build on the recently introduced SiN-based smart tip platform^16^ and now incorporate high-resolution focused ion beam (FIB) milling to achieve nanometer tip separation while maintaining a rigid connection through a thin silicon nitride (SiN) support. The combination of the mechanical stability provided by the SiN platform with high-resolution milling yields a unique and straightforward approach to the fabrication of scanning probes and their future use in double-tip STM.

## Results

**Fig. 1:**
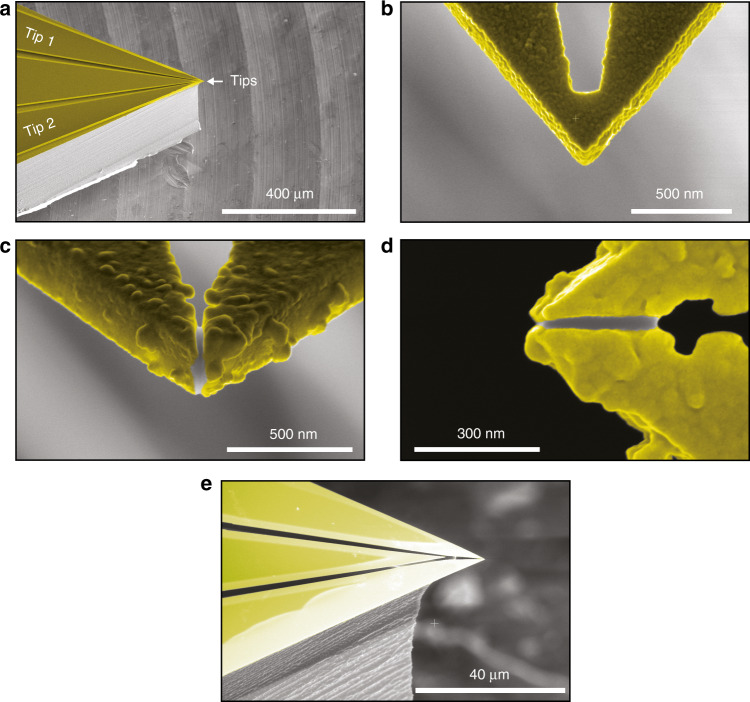
Colored SEM images of several double-tip devices after deposition of Au (yellow). **a** Tilted profile of the device showing the profile of the chip and the protruding tips. **b** Tip apex before FIB milling. **c** Tilted front view of the two tips after FIB milling. The two tips originated from a 55 nm gold layer, and the gap between the tips was ~35 nm; hence, the total distance between the points that were most likely to tunnel was ~50 nm. The required 3D alignment can be directly estimated from these tilted images. **d** Top view of the same device as (**c**) shows clear protrusion of the metal film beyond the SiN. **e** Side view of the device shown in (**c**) and (**d**).

Figure 1 gives an overview of several devices we fabricated using the methods described above. Figure 1a shows an overview of the chip, where the three contact pads separated by trenches can clearly be identified. The narrow bright lines along the pads are the regions consisting of undercut SiN covered in gold, like the rest of the chip. The angled view clearly shows the sidewalls of the chip and the overhanging tips where the contact pads meet. High-magnification images provide a detailed view of the apices of the tips, where Fig. 1b was taken before the FIB step. Such a device can act as a single-tip device or potentially be used to perform tip preparation through heating by running a current between the contacts, as suggested by Ciftci et al.^32^.

Figure 1c, d shows the apices of two tips from the front under a 52° angle and from the top, respectively. They allow us to clearly see the metal film coverage on the sides of the SiN layer as well as the separation between the tips, which was ~35 nm edge-to-edge. The relatively thick Au film became grainy, especially near the apex of the SiN, while it was smoother on the pads. Even though the coarseness of the metal made the exact location of the tip uncertain, the metal consistently covered the full apices (Fig. 1d). For this particular device, the FIB milling depth was set to 300 nm to ensure a cut through all the metal on the side of the SiN.

## Discussion

The general fabrication process herein follows the process for SiN-based smart tips introduced earlier for single-tip devices^16^. Here, we continue to build on this platform as we extend the fabrication procedure to create double-tip devices by incorporating several new techniques. The high-quality SiN that provides the base for the tips plays a central role in the design and fabrication of the devices. First, their excellent insulating, dielectric and mechanical properties provide the opportunity to keep the two tips mechanically attached while electrically separated. Second, the SiN also enables the use of a single lithography step that incorporates the additional complexity that the multiple tips and contact pads bring. By transferring the shape of both tips and their contact pads into the SiN, we can later use an isotropic silicon etch to create trenches that electrically separate the tips even after deposition of the metal, as we will show below.

### Pattern design

**Fig. 2:**
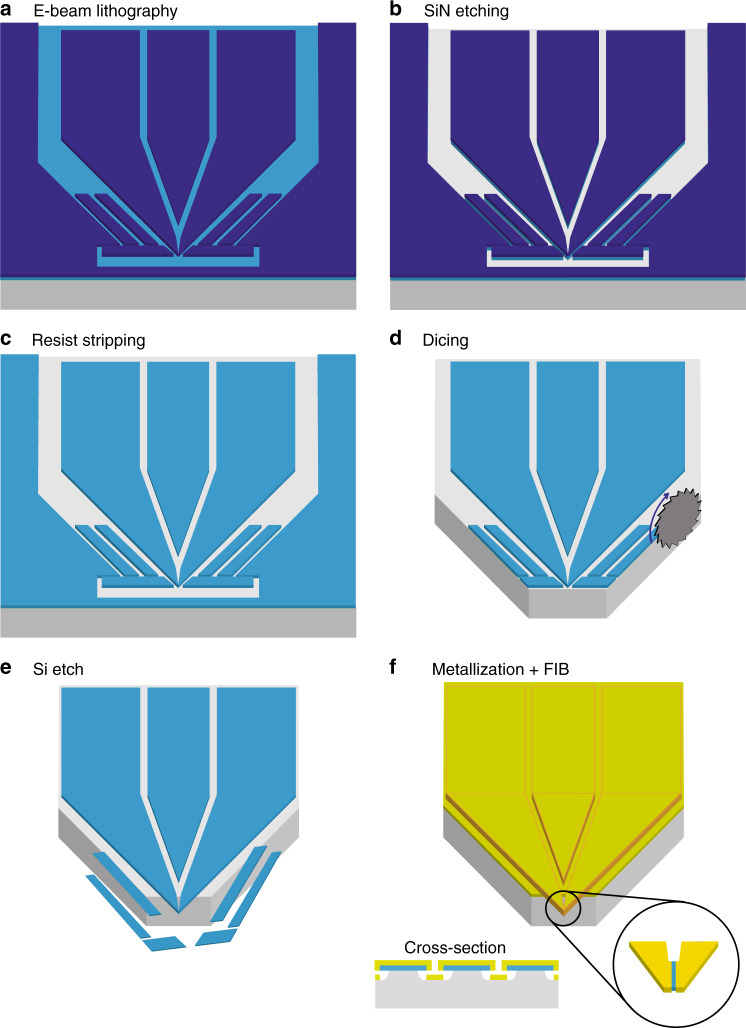
Fabrication procedure for double-tip devices. **a** The resist layer (purple) on the SiN (blue)-covered Si (100) chip (gray) is patterned by e-beam lithography, creating a large opening in front of the tips, around the contact pads, and along the lines that encircle the shields and the tips. **b** The pattern is then transferred into the SiN. **c** The resist is cleaned thoroughly, and a fresh layer of photoresist is applied to protect against debris from dicing (not displayed for clarity). **d** The chip is then diced (details can be found in Fig. 3). **e** After removal of the protective resist, the chip is undercut using an isotropic Si dry etch that removes the Si substrate primarily from the sidewalls causing the shields to drop off, leaving the tips freestanding. **f** We evaporate a Au layer on top of a Cr adhesion layer followed by focused ion beam milling to separate the two tips (inset right). The electrical isolation of the contact pad is illustrated in the cross-section inset.

A schematic overview of the full fabrication procedure is shown in Fig. 2. We start with a 200 nm thick layer of high stress silicon nitride deposited with low-pressure chemical vapor deposition (LPCVD) on both sides of a 200 µm thick Si (100) chip, and we deposit a 550 nm thick layer of ARP-6200.13 resist by spin coating on top for electron beam lithography. The pattern is created by a single exposure but consists of two parts. The first is the tip shape itself with the surrounding shields, which we include to minimize the overhang of the SiN surrounding the tip after the Si etching process, as described below. By covering these areas with the shields, the isotropic etch only affects the sidewalls and does not create a significant undercut from the top. The second part consists of the two contact pads and a center pad that is used to reduce the exposed area to shorten the exposure time and minimize the proximity effects of the exposure near the two tips. For future applications, this part can also act as a third contact pad (combined AFM/STM systems often include three electrical contacts). The large areas are exposed by a 40 nm electron beam with a beam step size of 20 nm and a dose of 400 µC/cm^2^, and the small structures are exposed by a smaller beam with a size of 18 nm, a step size of 2.5 nm and a dose of 320 µC/cm^2^, both with a 100 kV beam. Note that the narrow trenches between the tips/contacts could lead to electrical shorts formed by accidental left-over traces of resist inside the trenches or small pieces of dirt that connect the pads after deposition of the metal. Therefore, we enlarge the spacing between the tips and pads as we move away from the apices of the two tips that are still connected at this stage.

We then developed the chip in pentyl acetate (1 min) and MIBK:IPA 1:1 (1 min) followed by an IPA rinse (1 min), resulting in Fig. 2a. The pattern, consisting of the tip shape and six shields, is transferred into the SiN layer using a CHF_3_ etch for 5 min (Fig. 2b). After we start the removal of the resist by exposing the chip to an O_2_ plasma for 10 min, we then continue cleaning the chip by successive immersions into N-N-dimethylformamide (DMF) for 10 min, a positive resist stripper (PRS) for 10 min, followed by a boiling piranha solution at 135 °C for 8 min to remove all traces of the resist and other organic contaminants (Fig. 2c).

### Dicing

To bring the tip to the edge of the chip, we proceeded with dicing (Fig. 2d). Here, we prefer dicing over a through-wafer deep reactive ion etch (DRIE) since it allows us to easily explore different designs with each new iteration and reduces the number of fabrication steps. We would, however, like to stress that our method is fully compatible with full wafer processing, as we discuss later.

**Fig. 3:**
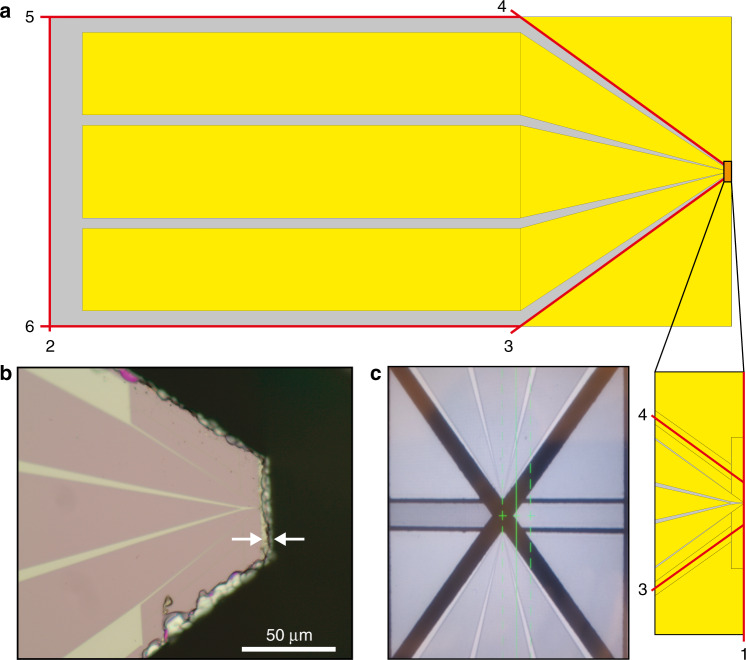
Double-tip device design. **a** Double-tip pattern design with dice lines shown in red. The gray areas indicate Si, while the yellow parts are covered with SiN. The small features, such as the tips and the surrounding shields, are highlighted in the inset on the bottom right. **b** Optical microscopy image of a chip after dicing. The silicon (gray) left after the dicing extends up to 6 µm beyond the SiN (pink). **c** Screen capture of a chip containing two opposing smart tips after dicing as seen through the microscope on the dicer. For illustrative purposes, the image is taken at low magnification, and the alignment of the blade is performed at high magnification. The black areas are diced.

Before the dicing step, we first protect the chip surface against any residual debris by applying a new layer of photoresist. From a typical 10 × 10 mm^2^ chip, we cut two smart tips to ensure that the small features of the tip are in the center of the chip for optimal resist conditions for EBL (Fig. 3c). Successful dicing results in (i) smooth sidewalls, such that the overhanging tip is the most protruded feature, (ii) there is minimal chipping of the Si and, (iii) there is good alignment accuracy.

The first requirement is obtained by choosing the optimal blade and settings for the dicer (Disco dicer DAD 3220). The Disco ZH05-SD2000-N1-90 blade at a forward speed of 3 mm/s yields optimal results in terms of chipping and sidewall smoothness. The limited residual roughness on the sidewall is further smoothed out by the isotropic Si etch described in the next section.

To align the dice consistently down to a few microns from the patterned tip, we minimize the amount of chipping of the Si along the dice line (Fig. 3b). The blade is therefore typically dressed – resharpened by cutting into a special substrate and increasing the exposure of the diamonds in the blade for each new chip (two tips), and we perform the most critical dice (number 1 in Fig. 3a) first, as the chipping increases with each dice.

Last, to align accurately to the patterned tips, we calibrate the width of the blade with a so-called hairline adjustment.

### Suspending SiN tip

First, we wash away the residue created by dicing with the removal of the protective photoresist layer, and then the chip is cleaned again with a 10 min O_2_ plasma. After these cleaning steps, we isotropically remove part of the Si substrate by exposure to an SF_6_ plasma for 3–4 min inside an inductively coupled plasma (ICP) etcher. Reactive ion etching is often a combination of chemical reactions, where F^−^ ions react with Si, and ion bombardment, where the ions are accelerated by a bias into the sample, which removes material. Here, however, to prevent any anisotropy in the etch, we apply a bias except for during a short ignition pulse *<*1 s to ignite the plasma, resulting in a predominantly isotropic Si etch^33^. To increase the selectivity of the SiN over the Si, the chip is cooled to *−*50 °C.

### Metallization

After the overhang is created, we proceed to deposit the metal film. While our previous work^16^ relied on sputtered Au films to ensure maximum coverage of the SiN sidewalls, here we use an electron beam evaporator to deposit a 3–5 nm Cr adhesion layer and a 45–60 nm thick Au film. The evaporation of Au films is more directional but should in combination with a Cr layer underneath show improved adhesion to the SiN. To counter the effects of the directionality of the evaporation process compared to that of sputter deposition, we place the chip at an angle of ~40° and rotate it at 10 rpm to obtain an appropriate sidewall coverage.

### Optimizations and challenges

**Fig. 4:**
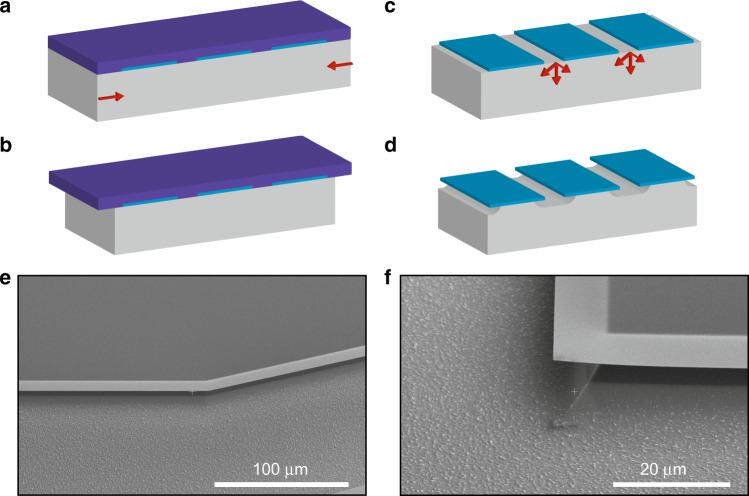
Procedure for minimizing trench depth and width between the contact pads (blue). A layer of photoresist (purple) covers the full chip (**a**), and we etch only the sides of the chip, creating a tip overhang (**b**). The resist is then removed (**c**), and an additional very brief isotropic Si etch is used to create the trenches between the pads for electrical separation (**d**). SEM images of the finished device showing the undercut along a trench (**e**) and at a corner (**f**).

Before demonstrating the final devices, we employ the optional optimization. If the overhangs created on all edges break, shorts to the lower lying metal on the Si can occur, especially during mounting in the STM tip holder. To date, both the protrusion of the tips and the overhanging sides of the contact pads have been created with the same isotropic Si etch. We do this after dicing and subsequent removal of the protective photoresist with acetone and IPA. However, we can also choose to leave the resist used during dicing on and perform the etch, as illustrated in Fig. 4. Now only the sidewalls of the chip retract while the top layer is protected, therefore we create a protrusion of the tip, but no trenches between the contact pads. The latter, however, is needed to create electrical separation of the pads. Once the tip overhangs and (almost) all shields are held by the resist, we spray acetone and IPA to clean off the resist in the direction away from the tip to prevent the shields from landing on top. Finally, we can perform a very short additional isotropic etch to form the contact pads. The result is smaller overhanging regions around the sides of the contact pads. The decrease is determined by the length of the last etch.

### FIB milling

The minimum tip-to-tip distance previously achieved by having two separate SiN tips is 45 nm for a test device using 50 nm SiN and (sputtered) 20 nm Au^16^. Here, we work with a thicker and more reliable SiN film and Au layer. This also enlarges the radii of curvatures of the two tips and thereby limits the tip-to-tip distance to more than twice the radius, which is a similar limitation to bringing two individual tips into close proximity. The use of a 50 nm thick SiN and a very thin film reduces tip separation but causes uncertainties. First, depending on the length of the overhanging tips, the thin SiN tends to bend upwards due to the intrinsic stress of the film. While this bend is not a problem in principle, it might not always be equal for both tips when not connected. We therefore decided to use thicker 200 nm SiN in combination with a slightly thicker evaporated Au film.

As a result of the excellent control we have over the shape of the tips, we create devices where the two apices are joined together at both the SiN and Au film and separate the tips using FIB milling. The aim is to mill only the metal layer and keep the SiN attached. A priori, this method presents several advantages: (i) FIB milling has the ability to achieve a higher resolution than EBL^34^, especially when combined with a 200 nm deep etch through the SiN; (ii) the tip separation is not determined by the film thickness; (iii) the mechanical connection formed by the SiN eliminates possible height or tip-to-tip distance fluctuations, compared to those for earlier reported proof-of-principle devices^16^; and (iv) this approach improves the overall (macroscopic) stability of the tips. The dielectric breakdown voltage that can result in a short circuit between the two tips should occur well above 5 V for these structures and should not hinder normal STM operation. Another possibility for the separation of the metal we did not explore involves the use of electromigration to create a tip separation to possibly smaller than 5 nm^35^, although sufficient control over where the separation occurs might be challenging.

A well-known side effect of FIB milling is the addition of Ga^+^ ions a few nanometers into the exposed top layer of the material. The impact of the ions can result in lattice defects, incorporated Ga, and heat^34^. The range of added Ga^+^ ions can vary from 10 to 100 nm in depth and 5 to 50 nm in width. The Ga^+^ ions at the Au tip surface may be a cause for concern if they have an effect on the quality of the STM tips or their electronic properties; however, previous STM experiments have used tips modified by FIB before^13,36^. The difference here is that redeposition of the insulating SiN can occur. However, a short immersion in an HF solution or exposure to vapor HF^37^ can be used to remove a few nanometers of SiN without damaging the overall structure of the tips. Furthermore, a common in situ tip preparation technique called mechanical annealing^38,39^ allows us to pick up clean gold from a Au (111) surface and may assist in attaining a clean STM tip and should assure tip sharpness. Variation of the tip-to-tip distance due to the graininess of the metal film may still occur but can be readily improved by optimization of the evaporation temperature; the use of thinner, less grainy films, such as AuPd; and additional shaping of the tip using FIB.

### Tip characterization

To validate the fabrication method described in the paper, we test the device in a modified scanning tunneling microscope (STM) manufactured by RHK.

**Fig. 5:**
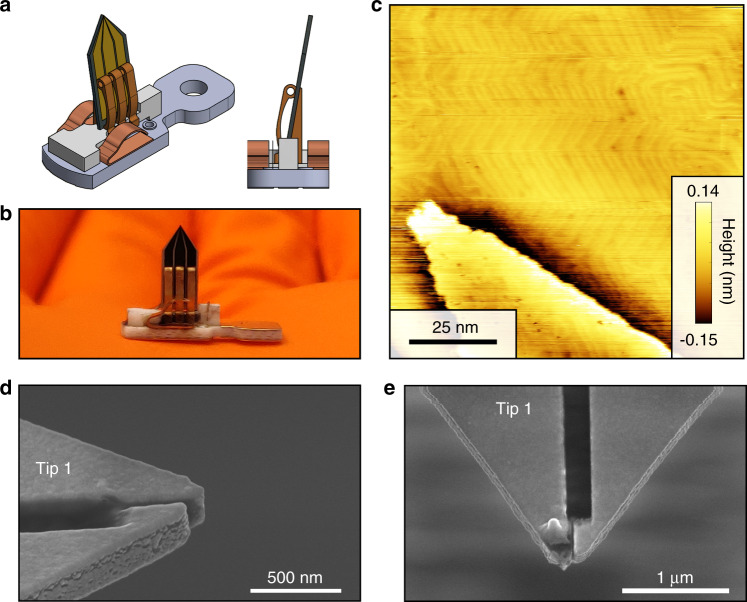
Testing a fabricated device on a Au (111) surface. **a** Schematic drawing of a device holder including the double-tip device. **b** Photograph. **c** Topographic image of gold on a mica sample using our tip for STM imaging taken with setup conditions where *V*_*b*_ = 0.75 V and *I* = 50 pA. **d**, **e** SEM micrograph of the device used to measure (**c**) before and after applying a voltage of >5 V between the two tips.

We built a custom holder for the device (Fig. 5a, b) consisting of three phosphor bronze contact clamps attached to an Al_2_O_3_ block on a base plate made out of a special printed circuit board equipped with vias to contact the STM. The device is then inserted into the holder and held by the contact springs. We insert the holders into our cryogenic ultrahigh vacuum STM.

For the imaging and breakdown experiments, we prepare a gold film with three cycles of Ar^+^ sputtering. (0.75 kV, 3 mA at 5 × 10^−5^ mbar for 15 min) and subsequent annealing at ~390 °C for 30 min under UHV conditions. We then scan the gold on the mica sample. Figure 5c shows a topographic image from the Au(111) surface exhibiting both step edges and typical herring-bone reconstruction. A gold surface is commonly used to assess tip quality and stability; however, since it is metallic, gating effects from the second probe are not expected, as the potential drop occurs completely between the tips and sample. Finally, we investigate the breakdown voltage between the tips by applying voltages of 1 V, 2.5 V, 5 V, and 7.5 V between the tips. The voltage is increased with the tip retracted, and the device is then tested on the Au (111) surface with the voltage fixed. The device continues to function up to 5 V applied between tips, but breaks down at 7.5 V, as confirmed by SEM images (see Fig. 5d, e).

## Methods

### Dicing

The full dicing procedure is as follows. First, we dress the blade, load the chip and proceed to make a first cut and perform the hairline adjustment. Then, with a sequence of individually aligned dices as indicated in Fig. 3a, c, we cut out the chip shape. Line 1 is the one close to the tips and is therefore performed first. Line 2 follows and maintains the same alignment to ensure that when the chip stands upright, the bottom is exactly parallel to the top where the tips protrude. Cross lines 3 and 4 run through the shields typically around 20 µm away from the tip on each side (Fig. 3b). Finally, we dice lines 5 and 6 parallel to each other and set the width of the chip to 3 mm.

### FIB milling

For the milling procedure, we load the chips on a piece of carbon tape into the FIB system with the sample holder firmly screwed in to prevent small displacements during tilting. We adjust the focus of the electron beam repeatedly as we move the stage to its working distance. We also correct for stigmatization and ensure that the electron beam is not shifted with respect to the ion beam for the alignment procedure. Then, we pick a reference point at high magnification and tilt the sample 52° towards the ion beam while imaging with the electron beam. As soon as we image with the ion beam, the milling starts; therefore, we first find an area close to the tip but keep it outside the field of view. We can set up such that the fields of view of the electron and ion beam are the same and the magnifications are linked. Before we mill, we focus the ion beam at ×100,000 magnification. Using the electron beam, we navigate to the tip, take a single scan with the ion beam and align the milling path. After the alignment, we typically take a second scan to check and compensate for a slight drift in the image. Then, we perform the milling process over a length from 1 to 2 µm in less than a second using an optimized recipe for Au with a milling depth from 200 to 300 nm.

## Conclusion

We demonstrated a new fabrication procedure for integrated double-tip devices with a tip separation of ~35 nm based on our highly flexible SiN-based smart tip platform. By etching the contact pads and two tip geometry into the SiN layer, we can create double-tip devices with an easy-to-implement process. The excellent mechanical and insulating properties of the SiN allow us to keep the tips attached via the SiN and utilize high-resolution FIB milling of the metal layer to separate the two tips. This leads to increased mechanical stability, better alignment of the tips with respect to each other and removal of the need for any electromechanical actuation^40^.

The integration of these devices in existing commercial STM systems is straightforward when multiple tip contacts are available. The chips are only 200 µm thick, can be made in various sizes and include large contact pads, making them well suited for the limited space available inside an STM head. Another benefit for the routine use of these tips is the ability to upscale the production of the devices to full wafer processing. By replacing the dicing of individual chips with a through-wafer Bosch etch, one could make a large number of chips from a single 4” wafer. For this, we would use a SiO_2_ hard mask combined with an electron beam or optical lithography to align the chip pattern to the underlying SiN tips/contacts with possibly even better accuracy compared to that of dicing. Once optimized, this is expected to yield faster, larger and more consistent production than current approaches.

A fully functioning double-tip STM based on the tips presented here differs significantly from a multiprobe system using metal wire tips. Here, the tips are joined at a fixed distance from each other on a chip that moves via a single piezoelectric tube positioning system. Consequently, the control over the two tips has unique challenges. To instigate tunneling in both tips, tilt stages to compensate for the slight difference in the length between the tips and a sample tilt are required. Based on the measurement of 4 different double-tips, we conclude that the typical angles between tips in various devices range from 4 to 15 degrees. After pretilting the sample or the devices, the final alignment for a double-tip experiment can be performed with a single piezoelectric element. As the 3D alignment is known from SEM images (see Fig. 1), one tilting stage is sufficient, while two stages would further increase the flexibility. Naturally, this results in a more advanced feedback scheme that takes the added tilt stage and both currents into account. Without a compact tilt stage, devices can be used in single-tip operation where the second tip, which is not tunneling, can be used as a gate to locally change the carrier density of dilute electron systems, such as semiconductor nanostructures^28^ or underdoped Mott insulators.
